# Comparing Characteristics of Adverse Drug Events Between Older and Younger Adults Presenting to a Taiwan Emergency Department

**DOI:** 10.1097/MD.0000000000000547

**Published:** 2015-02-20

**Authors:** Yen-Chia Chen, Hsien-Hao Huang, Ju-Sing Fan, Min-Hui Chen, Teh-Fu Hsu, David Hung-Tsang Yen, Mu-Shung Huang, Chien-Ying Wang, Chun-I Huang, Chen-Hsen Lee

**Affiliations:** From the Department of Emergency Medicine (Y-CC, H-HH, J-SF, T-FH, DH-TY, M-SH, C-YW, C-IH, C-HL), Taipei Veterans General Hospital; Institute of Environmental and Occupational Health Sciences (Y-CC), School of Medicine, National Yang-Ming University, Taipei, Taiwan; Rocky Mountain Poison and Drug Center (Y-CC), Denver Health, Denver, CO, USA; Institute of Emergency and Critical Care Medicine (H-HH, J-FS, T-FS, DH-TY), School of Medicine, National Yang-Ming University, Taipei, Chin-Kang Clinic (M-HC), New Taipei; and Department of Emergency Medicine (M-SH, C-YW, C-IH, C-HL), Faculty of Medicine, School of Medicine, National Yang-Ming University, Taipei, Taiwan.

## Abstract

To compare the proportion, seriousness, preventability of adverse drug events (ADEs) between the older adults (≥65 years old) and younger adults (<65 years old) presenting to the emergency department (ED), we conducted a prospective observational cohort study of patients 18 years and older presenting to the ED. For all ED visits between March 1, 2009, and Feb 28, 2010, investigators identified ADEs and assessed cases using the Naranjo adverse drug reaction probability scale. Outcomes (proportion, seriousness, and preventability of ADE, length of ED stay, and hospitalization) and associated variables were measured and compared between younger and older adults. The results showed that of 58, 569 ED visits, 295 older adults, and 157 younger adults were diagnosed as having an ADE and included in our analysis. The proportion of ADEs leading to ED visits in the older group, 14.3 per 1000 (295/20,628), was significantly higher than the younger group of 4.1 per 1000 (157/37,941).The older group with ADE had a longer ED stay (odds ratio [OR] 3.5, 95% confidence interval [CI] 1.9–6.4 for stay ≥ 24 hours) and larger proportion of preventable ADEs (OR 2.2, 95% CI 1.4–3.6) than the younger group, but there was no significant difference in terms of serious ADEs (OR 0.6, 95% CI 0.3–1.3 for fatal and life threatening) and hospitalization (OR 1.5, 95% CI 0.9–2.6) between the 2 groups. In addition, patients in the older group were more likely to be male, to have symptoms of fatigue or altered mental status, to involve cardiovascular, renal, and respiratory systems, and to have higher Charlson comorbidity index scores, higher number of prescription medications, and higher proportion of unintentional overdose. In conclusion, the proportion of ADE-related ED visits in older adults was higher than younger adults, and many of these were preventable. The most common drug categories associated with preventable ADEs in the older adults were antithrombotic agents, antidiabetic agents, and cardiovascular agents.

## INTRODUCTION

Adverse drug events (ADEs) are a substantial cause of emergency department (ED) visits and a major health care concern.^1^ They impair patient health and increase health care cost.^2^ Prior studies estimated that the prevalence of ADEs leading to ED visits varied among different age groups, with older adults (4.9 per 1000) experiencing the highest occurrence compared with adults (2.0 per 1000) or children (2.0 per 1000).^3,4^ Older patients are more likely than younger patients to have multiple diseases (which will increase the susceptibility to adverse drug effects) and take more medications (which increases the probability of adverse drug effects). Therefore, polypharmacy and the disproportionate use of medications, combined with age-related pharmacokinetic and pharmacodynamic changes, place older adults at higher risk for medication-related problems and ADEs.^5–7^

Age-specific differences, particularly in pediatric (<18 years), adult (18–64 years), and elderly (≥65 years) populations, may reflect age-related differences in patients and medications. Tache et al^8^ reported the top 3 drug categories associated with ADEs in the adult group (cardiovascular, anti-infective, and analgesic drugs) were different from those in the older adult group (cardiovascular, anticancer drugs, central nervous system drugs). Dormann et al^9^ also demonstrated that compared with the younger patients, a higher proportion of elderly patients had ADEs that were considered preventable (28.4% vs 65.7%, *P* < 0.001). However, some studies did not show that age increased the risk of ADEs.^10^ The study by Gomes et al mentioned that the elderly patients did not appear to have higher risk of developing drug allergy and that there was no increase in the severity of allergic reactions or drug-related mortality.^11^ In the literature, information on whether age-related differences reflect differential patterns of ED-care utilization throughout the life cycle is limited. Furthermore, age-related characteristic differences in ADEs, age-associated drug types, and other clinical profiles were not clarified. Therefore, the age-based patient data may be of importance to help with delineating the characteristics of ADEs leading to ED visits and to prevent possible ADEs and improve patient safety as a whole.

To better understand the differences in clinical profiles and proportion of ADEs leading to ED visits between the older and younger adults, we conducted a prospective cohort analysis of patients to compare the proportion, seriousness, preventability, and characteristics associated with ADEs between the 2 age groups at a tertiary medical center in Taiwan.

## METHODS

### Study Design and Setting

This was a prospective cohort study enrolling adult ED patients from February 2009 through March 2010 at Taipei Veterans General Hospital, a tertiary referral center in northern Taiwan. The study protocol was approved by the Institutional Review Board of the hospital.

### Selection of Participants

Using the definition by Nebeker et al,^12^ “an adverse drug event is an injury resulting from the use of a drug,” the term ADE includes harm caused by the drug, such as adverse drug reactions (ADRs) and overdoses, as well as consequences from using the drug, such as the need for dose reductions and discontinuations of drug therapy. In our study, ADE cases were defined as patients who developed adverse effects such as ADRs and overdoses caused by drugs, as well as consequences from using the drug which led to ED visits^12,13^ and the need for dose reductions and discontinuation of drug therapy (eg, bradycardia in a patient taking β-blockers). In order to better define ADE cases and minimize the possibility of underdetecting or underreporting of ADE, the Naranjo scoring system^14^ was adopted to classify the probability of ADE, and structured instructions were announced to enhance the collaboration among ED physicians and relevant subspecialists for identification of ADE cases, as described in detail in our previous publication.^1^

Events that were not considered ADEs included those lacking temporal relationship between drug administration and clinical symptoms, therapeutic failures, drug withdrawal syndromes, and follow-up visits for previous ADE. Patients were also excluded if a drug was taken for other than ordinary therapeutic purposes, such as suicide attempts or recreational uses. Patients aged 18 and older registered at the study hospital were asked to provide relevant information on drug name, dosage, method of administration, and length of therapy prior to the ED visit. Drugs in this study were categorized as prescription drugs, over-the-counter drugs, vaccines, vitamins, and nutritional supplements. Liquors, alcoholic beverages, tobacco products, illegal substances, and topical cosmetics were excluded.

### Data Collection and Processing

Using a standardized data collection form, the following information was obtained from interviews with patients and/or caregivers as well as medical charts: demographic data, gender, age, clinical history, clinical symptoms, laboratory tests, treatments, and clinical outcomes. Individual event count was registered for each occurrence of clinical symptoms, signs, systemic complications, and death during the ED presentation. All the data collection forms were kept and transcribed into the study database. We used the International Classification of Disease 9th revision (ICD-9) (World Health Organization's Ninth Revision, International Classification of Diseases) to classify the diagnosis and any associated diseases during the ED visits. The Anatomical Therapeutical Chemical (ATC) system was applied to categorize the drugs. Disagreement regarding the culprit drug was resolved by research team discussions.

### Outcome Measures

The primary outcome measures were the proportion, seriousness, and preventability of ADEs. The probability that a drug caused the ED visit was assessed using the World Health Organization (WHO) classification (certain, probable/likely, possible, and unlikely). The seriousness of ADE was graded as fatal, life threatening, moderate (need to be treated), and mild (no need to be treated) per WHO definition.^15,16^ Preventability was categorized as preventable or not preventable.^17^ Preventable ADEs were defined as adverse drug effects related to improper prescribing, monitoring, or compliance, such as prescribing a high dose inappropriate for the patient's age or disease state and administering a drug to a patient with known hypersensitivity.^18^ The Charlson comorbidity index score was used to calculate and estimate the severity of comorbid disease.^19^ The secondary outcome measures included disposition after the ED visit such as hospitalization, length of ED stay, and drug category of ADEs.

### Data Analysis and Presentation

Independent *t* test was employed for the comparisons of continuous variables, expressed as the mean ± standard deviation (SD), and the Fisher exact test for categorical variables, expressed as the proportion in percentage (%). The seriousness of ADE was divided into binary outcomes: fatal and life threatening versus moderate and mild. The length of ED stay was categorized as ≥12 or ≥24 hours. Statistically significant variables (*P* < 0.05) identified in the univariate analysis were further entered in the multivariate model. Multivariate logistic regression was used to assess odds ratios between older and younger adults in terms of main outcome measures. Crude and adjusted odds ratios (ORs) were expressed plus 95% confidence interval (CI). All analyses were carried out using The Statistical Product and Service Solutions (SPSS for Windows, Version 19.0; SPSS Inc, Chicago, IL).

## RESULTS

**FIGURE 1:**
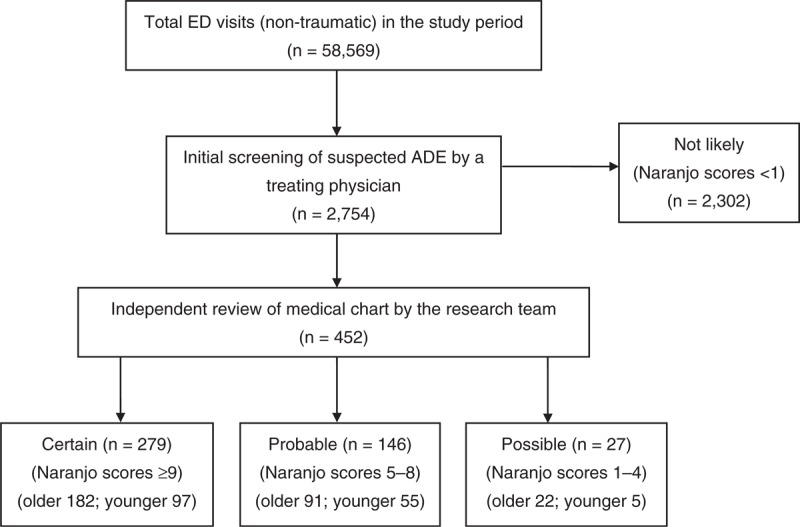
Algorithm for identification of ADEs in patients presenting to the ED. ADE = adverse drug event, ED = emergency department, Older = aged 65 years or older, younger = aged less than 65 years.

The study algorithm was shown in Figure 1. Patients were recruited from March 1, 2009, through February 28, 2010. A total of 58,569 nontraumatic patients presented to our ED during the study period. Of these, 452 cases (0.77%) with physician-documented ADEs were identified. (Figure 1)

The proportion of ADEs leading to ED visits in the younger group was 4.1 per 1000 (157/37,941) and 14.3 per 1000 (295/20,628) in the older group. Compared with the younger group, the patients in the older group with ADEs was more likely to be men (68.5% vs 46.5%), to have a higher mean Charlson comorbidity index scores (3.1 ± 2.1 vs 1.8 ± 2.1; mean ± SD), and to use a higher number of drugs (8.0 ± 3.9 vs 5.6 ± 3.9). Of the 125 ADEs related to drugs that require regular monitoring to prevent acute toxicity (antithrombotic agents, antidiabetic agents, anticonvulsants, digitalis, glycosides, theophylline, and lithium), the older group had a statistically significantly higher occurrence of ADEs than the younger group (32.2% vs 19.1%). (Table 1).

**TABLE 1:**
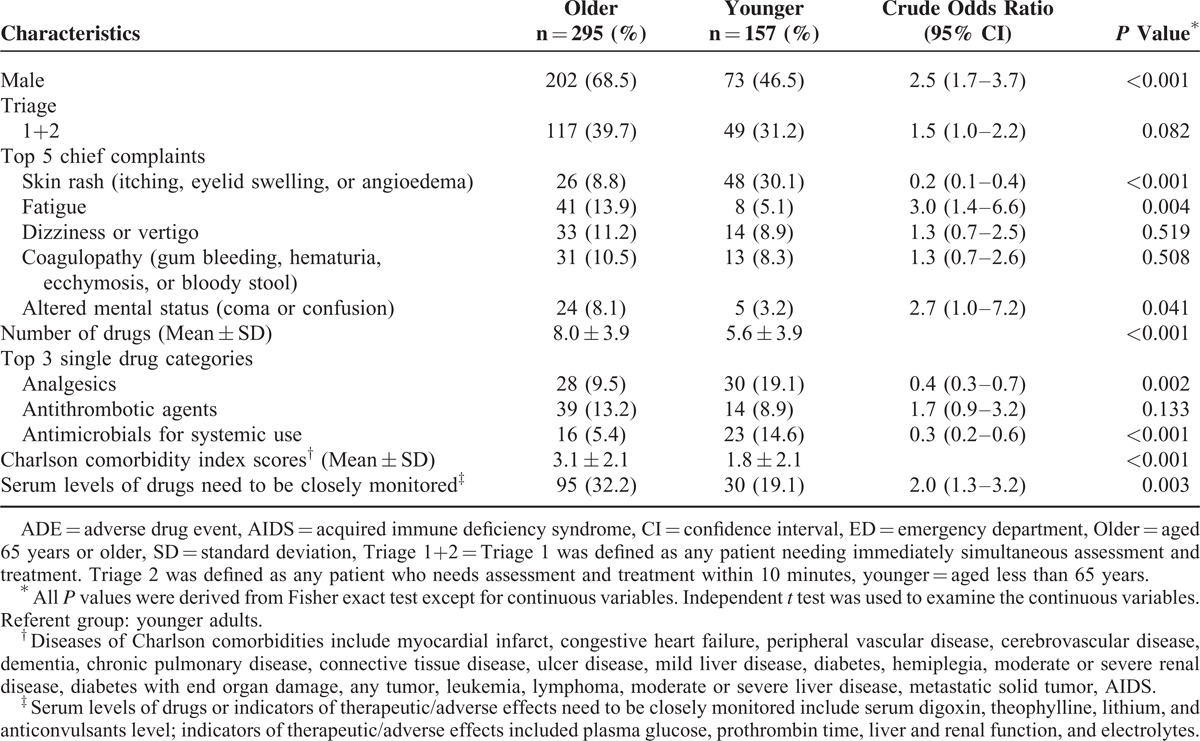
Distribution of Demographic and Clinical Characteristics of 452 Adults With ADE

Most ADEs (n = 401, 89%) were associated with a single drug. The remainder (n = 51, 11%) was associated with drugs from more than 1 therapeutic category. Overall, the most common categories of drugs associated with ADEs were analgesics (12.8%), antithrombotic agents (11.7%), and antimicrobials for systemic use (8.6%). The top 3 drug categories associated with ADEs in the older group (antithrombotic agents 13.2%, diuretics 10.2%, antihypertensives 9.5%, and analgesics 9.5%) were different from the younger group (analgesics 19.1%, antimicrobials for systemic use 14.6%, and antineoplastic agents 14.0%) (Table 2).

**TABLE 2:**
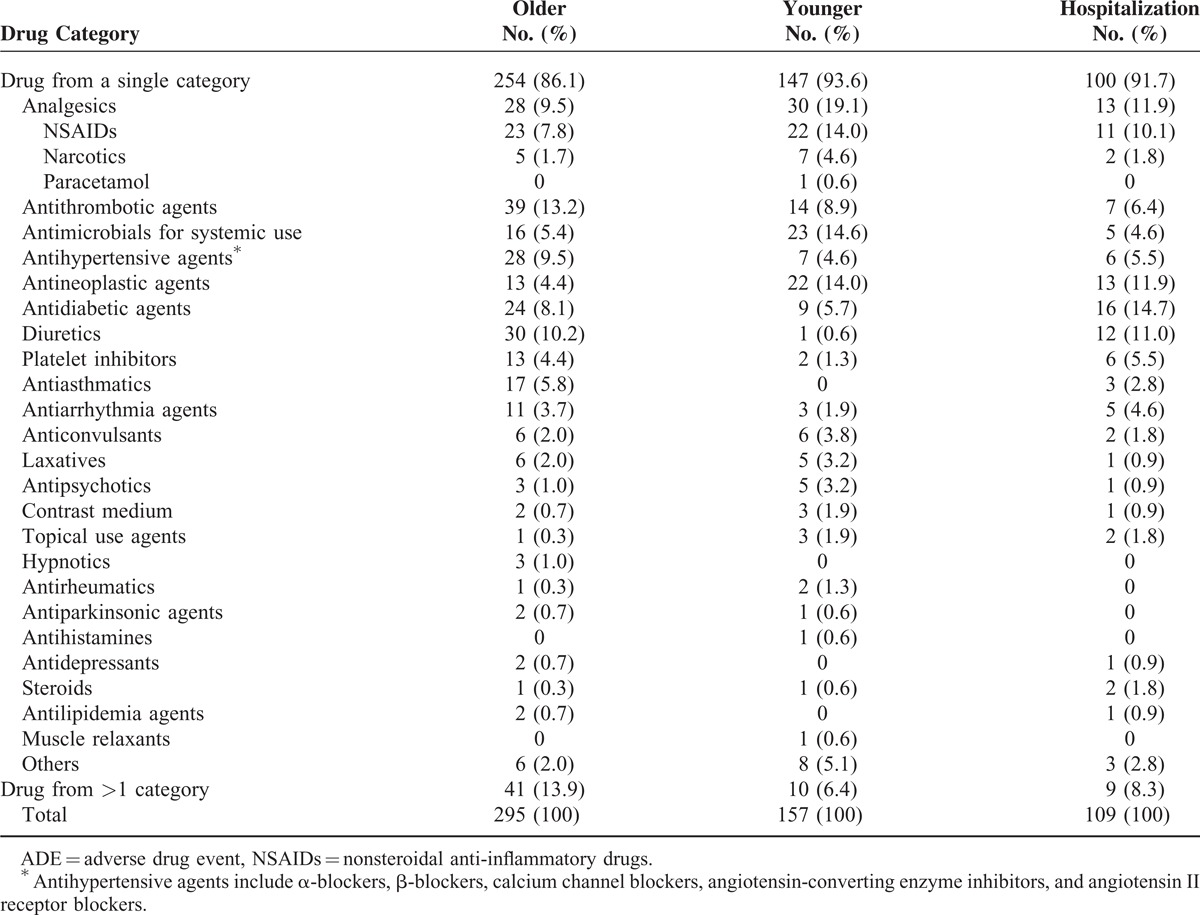
Drug Categories Associated With 452 Adults With ADE

Of the 452 patients with ADE, 8 (1.8%) cases were fatal, 37 (8.2%) were life threatening, 343 (75.9%) were moderate (required treatment), and 64 (14.2%) were mild (did not need treatment). Approximately 73% of the 452 ADEs were considered to be preventable. After controlling for the possible confounding effects of gender, Charlson comorbidity index scores, number of drugs, and serum levels of drugs or indicators of therapeutic/adverse effects needed to be closely monitored, we found that the older group had a longer ED stay (adjusted OR = 3.1; 95% CI 1.9–4.8 for stay ≥ 12 hours; adjusted OR = 3.5; 95% CI 1.9–6.4 for stay ≥24 hours), and a large proportion of elderly had preventable ADEs (adjusted OR = 2.2; 95% CI 1.4–3.6) compared to the younger group. In the multivariate models, there was no significant difference in terms of serious ADE (fatal and life threatening), ED treatment, and hospitalization between the 2 groups (Table 3).

**TABLE 3:**
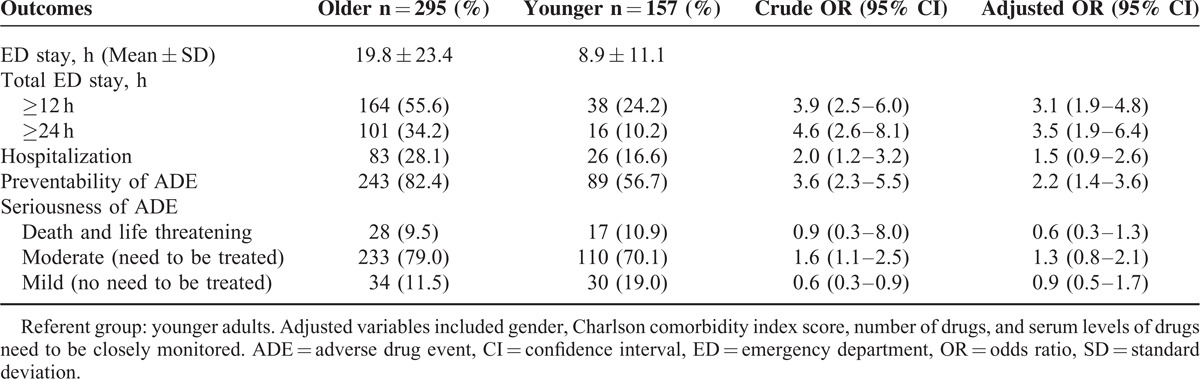
Outcomes of Older and Younger Adults With ADE

## DISCUSSION

Our investigation was the first prospective study in Taiwan to compare the proportion and characteristics of ADEs between the older adults and the younger adults presenting to ED. Our study found that patients aged 65 years or older were likely to have higher proportion of ADEs (14.3 per 1000 vs 4.1 per 1000, *P* < 0.001) and to have preventable ADEs and a longer ED stay. However, there was no significant difference in the incidences of serious ADEs and drug-related hospitalization between the older group and the younger group.

The proportion of ADE found in our ED (0.77% of all ED visits) is somewhat lower than the proportion reported in previous studies, which has ranged from 0.86% for ADRs to 3.9% for medication-related problems.^20–22^ This variability may be attributed to differences in study populations, methodology, and inclusion/exclusion criteria of ADE. Our result is similar to a study conducted in the United States using a national surveillance system (0.7% of all ED visits).^13^ Hohl et al reported that emergency physicians had suboptimal ability to identify drug-related adverse events of mild and moderate severity in ED.^23^ Therefore, another possible explanation for this lower proportion of ADE in our study could be related to underdetecting or underreporting of ADE by ED physicians.

While many ADEs are inevitable, we found that ADEs which occurred in the older group were more likely to be preventable than those in the younger group (82.4% vs 56.7%, adjusted OR = 2.2; 95% CI 1.4–3.6, *P* = 0.001). This finding was consistent with results from previous studies.^9,17^ A prior case-control study by Seeger et al reported that a strong association was noted among the drug dose, type of ADE, and preventability of ADEs, but allergic or idiosyncratic reactions were considered to be unpreventable.^24^ We found that medications requiring regular monitoring to prevent acute toxicity, such as antithrombotic agents, antidiabetic agents, certain anticonvulsants, digitalis, glycosides, theophylline, and lithium, were more likely to be associated with ADEs in the older group. This finding is in agreement with a similar ED-based study in Spain, which reported that the preventability of ADEs was related to drugs with a narrow therapeutic index.^25^ Therefore, it would be better to consider not only the prescribed medications for the elderly group but also the follow-up of these patients for medication monitoring as a strategy for preventing ADEs.

Our results showed that the older group had a longer ED stay than the younger group after controlling for the possible confounding effects of gender, Charlson comorbidity index scores, number of drugs, and serum levels of monitored drugs or indicators of therapeutic/adverse effects. One possible explanation for this is that the organ systems affected by the ADEs differed between the 2 groups. The older group had a significantly higher proportion involving the cardiovascular, renal, and respiratory systems (data not shown). These systems may require longer ED observation and treatment than cases involving the dermatologic system. Other possible explanation may be that age-related physiological changes in the older group may prolong the half-life of drug metabolism and clearance compared with the younger group,^26^ thus resulting in longer period of treatment and monitoring in ED.

In our study, ADEs occurring in the older group were most often associated with antithrombotic agents and diuretics, followed by cardiovascular agents and antidiabetic agents. These drug categories were similar to those of the preventable ADEs found in other studies involving the ambulatory setting^17,27^ In contrast, ADEs in the younger group were more likely to be related to analgesics and antimicrobial agents, which were known to be responsible for a variety of skin-related clinical presentations such as itching, eyelid swelling, or angioedema. Similar findings were reported in other studies.^13,28^ The difference is probably due to different imputable drug categories and category of ADEs in each group. Concentrating interventions on these drug category variations in different age groups could appreciably reduce the number of preventable drug-related ED visits.

Our study had some limitations. First, we did not calculate the study sample size in advance, and this study may possibly be statistically underpowered. Second, patients with ADEs might have been treated in other settings (eg, primary clinics, local hospitals, or other tertiary medical centers) and then transferred to our hospital for admission without ED evaluation. It is also possible that some ADE cases were undetected or unreported by involved physicians in ED. The above possibilities could most likely lead to underestimation of the actual occurrence of ADEs.^4^ Third, since some of the data were collected by patient interviews, our results may be affected by recall bias. Fourth, since our study was based in 1 single veterans hospital, it is possible that physicians prescribing and monitoring patterns specific to this hospital could have influenced the results. It is also possible that our results may not be generalizable to the rest of Taiwan. In addition, we have not prospectively validated these results, and some factors may be less predictive.

## CONCLUSIONS

In conclusion, the proportion and preventability of ADE-related ED visits may vary by age group. Compared to the younger adults, the older adults may have higher proportion of ADE-related ED visits. ADEs occurring in the older adults may be more likely to be preventable, especially those with antithrombotic agents, antidiabetic agents, and cardiovascular agents. Though we did not find significant difference in terms of serious ADEs and ADE-related hospitalization between the older and younger groups, further studies are needed to provide more information, which would help in the development of interventions aimed at improving the safety of prescribing medications and strategies for patient follow-up for drug monitoring and adherence to medications.
